# Traditional Chinese medicine manual therapy for adolescent idiopathic scoliosis: a case report

**DOI:** 10.3389/fped.2024.1500373

**Published:** 2025-01-21

**Authors:** Bowen Zhu, Miaoxiu Li, Jun Ren, Tianxiang He, Xin Zhou, Shoujian Wang, Lingjun Kong, Min Fang

**Affiliations:** ^1^ Department of Tuina, Shuguang Hospital, Shanghai University of Traditional Chinese Medicine, Shanghai, China; ^2^ Institute of Traditional Chinese Medicine and Tuina, Shanghai Research Institute of Traditional Chinese Medicine, Shanghai, China

**Keywords:** adolescent idiopathic scoliosis, manual therapy, traditional Chinese medicine, low back pain, case report

## Abstract

**Objectives:**

This case demonstrates the efficacy and safety of Traditional Chinese Medicine manual therapy (TCMMT) for the treatment of mild to moderate AIS.

**Methods:**

An 10-year-old girl who suffered from low back pain (LBP) with marked shoulder inequality and razorback postural abnormalities, with a clinical diagnosis of idiopathic scoliosis, and the Cobb angle of 20° and angle of trunk rotation (ATR) of the thoracic vertebral segment at 7°, and ATR of the lumbar vertebral segment at 8°, was treated with TCMMT. The patient initially recovered from centralized LBP with repeated TCMMT twice per week for 1 month. Subsequently, the frequency of TCMMT treatment was changed to 1–2 sessions weekly for 6 months. Before treatment, after the first TCMMT, 1 month and 7 months of treatment, and 18 months of follow-up, we used ATR or Cobb angle and health-related quality of life (HRQOL) for assessment. The HRQOL was assessed using the visual analog scale (VAS) scores and the Scoliosis Research Society-22 (SRS-22) patient questionnaire. The minimum clinically important difference (MCID) was used to assess the effectiveness of clinical measures based on a “responder analysis”.

**Results:**

(a) The patient's VAS score was 40/100 before treatment, 25/100 after the first treatment, 15/100 after 1 month of treatment, 12/100 after 7 months of treatment, and 15/100 at follow-up to 18 months. (b) The patient's SRS-22 score was 54/110 before treatment, 61/110 after the first treatment, 79/110 after 1 month of treatment, 106/110 after 7 months of treatment, and 104/110 at follow-up to 18 months. (c) Before treatment, the patient's thoracic ATR angle was 7° and the lumbar ATR angle was 8°, there was no change in the ATR angles of the thoracic and lumbar spine after the first treatment. The thoracic ATR angle was 6° and the lumbar ATR angle was 5° after 1 month of treatment. The thoracic ATR angle was 1.5° and the lumbar ATR angle was 3.5° after 7 months of treatment. The thoracic ATR angle was 2° and the lumbar ATR angle was 4° at the follow up till 18 months. (d) The patient's Cobb angle was 20° before treatment, 7° after the 7 month of treatment, and 8° at follow-up to 18 months. No adverse events during treatment.

**Conclusions:**

TCMMT is a conservative treatment option worthy of consideration when considering a conservative treatment strategy for AIS.

## Introduction

Scoliosis is a complex three-dimensional spinal deformity characterized by lateral deviation and vertebral body rotation, with idiopathic scoliosis (IS) being the most common form. Among adolescents, females have a higher prevalence of IS compared to males (1). It is estimated that 1%–3% of adolescents aged 10–16 years exhibit varying degrees of spinal curvature (2). In China, the incidence of adolescent idiopathic scoliosis (AIS) is reported to be 5.14% (3). Progressive AIS cases have been observed to demonstrate anterior spinal overgrowth that is disproportionate to longitudinal growth (4, 5). The etiology of AIS remains uncertain, with potential contributing factors including mechanical, metabolic, hormonal, neuromuscular, growth, and genetic abnormalities (6). Despite initially subtle symptoms, AIS can significantly disrupt bone development in adolescents, altering spinal mechanics and adversely impacting both physical and mental well-being. The primary objective of AIS management is to impede curve progression and circumvent the need for surgical intervention (7). Correction of AIS is particularly important, and the early development and implementation of effective and safe interventions for patients with AIS is of great clinical value. Addressing the high financial burden of AIS treatment and exploring novel therapeutic avenues are key areas of research focus in this field (8).

In recent years, with a significant increase in chiropractic studies focusing on the manual treatment of AIS, the stability and recognition of its efficacy have also notably improved. Manual therapy (MT) may be a more conservative treatment option, especially for children who choose to avoid brace therapy as much as possible and are not satisfied with regular observation of their progress (9, 10). In China, traditional Chinese Medicine manual therapy (TCMMT) encompassing soft tissue relaxation techniques and joint adjustment manipulation, is commonly employed for managing AIS. In our previous meta-analysis (11), we concluded that MT should be considered as a complementary and alternative therapy for the effective management of IS patients, especially as it had shown benefits in improving their pain and psychological well-being. The long-term efficacy of treatment of AIS is an important indicator to determine the stability of curative effect, but unfortunately it has been poorly reported, even though TCMMT is a common approach in clinical practice in China (12). For this, we describe a typical case of AIS treated with the TCMMT, aiming to report the long-term efficacy and safety of AIS and to provide physicians with additional therapeutic decisions in the conservative management of scoliosis.

## Case presentation

### History

A 10-year-old female patient exhibited progressive AIS characterized by a lumbar structural main curve. At the initial visit the patient's height was measured at 136 cm and weight at 31 kg, with no evidence of menarche or sexual development. The chief complaints were chronic low back pain (LBP) persisting for a duration exceeding six months following prolonged periods of sitting, and exacerbation of symptoms following strenuous physical activity. The physical examination revealed the presence of scoliosis, specifically a left curve of the lumbar segment, unequal shoulder height (left lower than right), and unequal length of both lower limbs (left longer than right). The Adam's Forward Bend Test (Adam's test) was operated by doctor who performed the assessment as follow: the patient's legs were erect, feet shoulder-width apart, and hands were folded together as they bent forward to a 90° bow, and the examiner used a scoliometer from the rear to review the patient's back for asymmetrical protuberances and assessed higher protuberances with a scoliometer measurement. The Adam's test of patient was positive, with the angle of trunk rotation (ATR) of 7° in the thoracic vertebral segment and 8° in the lumbar vertebral segment. Anteroposterior radiographs of the entire spine indicated AIS, with a Cobb angle of 20° in the lumbar vertebrae, vertebral rotation, and pelvic inclination. The Risser sign was Grade 0–1.

## Method

### Intervention

Before the first treatment, we provided patients with health education to clarify the treatment goals of AIS including fully understanding the occurrence, development and prognosis of AIS; correcting bad living habits, such as avoiding crossing legs, correcting improper sitting posture; changing their exercise habits, actively participate in sports activities for muscle building, and avoid competitive unilateral limb sports, etc. The detailed health education content was in Appendix 1.

**Figure 1:**
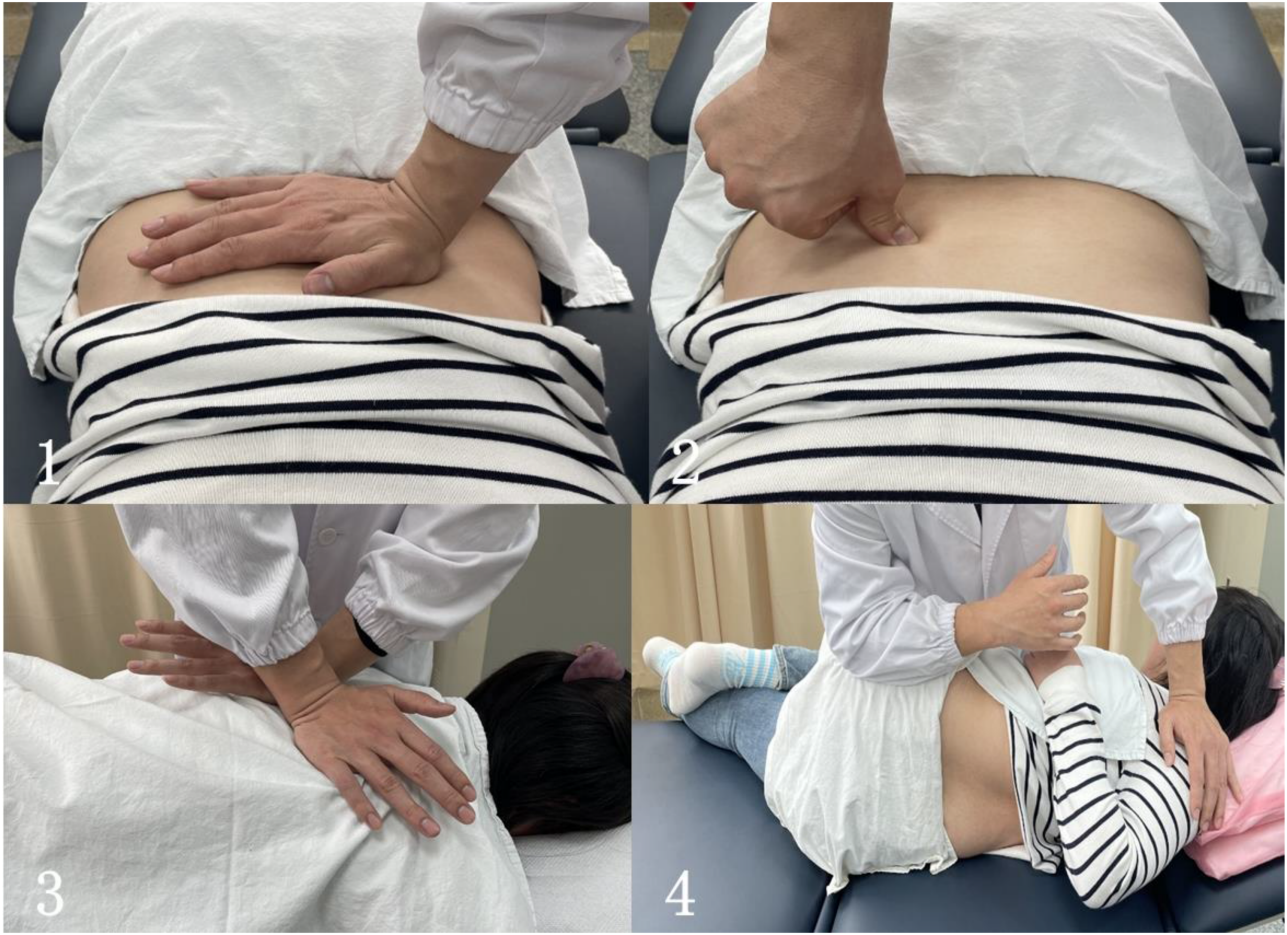
The operation process of TCMMT. **(1)** The soft tissue relaxation techniques in the convex side of paraspinal muscle ridges; **(2)** the soft tissue relaxation techniques in the paraspinal muscle contracture depression; **(3)** the journal joint adjustment manipulation for lateral bending of the thoracic vertebrae; **(4)** the journal joint adjustment manipulation for lateral bending of the lumbar segment (the subject in the figure was healthy individual and not the case patient reported in this study).

The treatment plan which the patient received was recommended by the clinician with reference to the scoliosis treatment guidelines. The patient eventually gave up regular observation. The TCMMT was performed by an experienced doctor with over 10 years of clinical practice, ensuring the quality and efficacy of the intervention. The operation process of TCMMT contained two parts: soft tissue relaxation techniques and spinal joint adjustment manipulation. (a) Soft tissue relaxation techniques: The Finger TPS Wireless system of the American PPS company was used to control the quality of the strength and frequency of the manipulation before treatment. The data acquisition frequency was 0.024 S time. The force parameters of the control TCMMT manipulation averaged 45 N, and the frequency was 2 Hz. The patient was in a prone position, and the physiotherapist stood beside the patient. On the convex side of paraspinal muscle ridges, press kneading was used to release the high-tension muscle group. In the paraspinal muscle contracture depression, thumb plucking was used to activate muscle activity. This operation lasted about 10–15 min. (b) Spinal joint adjustment manipulation: For lateral bending of the thoracic vertebrae, the doctor pushed the thenar against the convex side of the vertebral body at the top of the lateral curve with one hand, and the hypothenar against the opposite side of the upper vertebral body (concave side) with the other hand to form a cross. The patient was instructed to take a deep breath. When the patient was at the end of expiration, the doctor rotated her hands and pressed down vertically. Each stage of thoracic scoliosis was performed for this operation following the rhythm of the patient's breathing. For lateral bending of the lumbar segment, the patient was placed in the lateral position (convex side up) with bent the upper leg and straightened lower leg straight. The doctor stood in front of the patient, pushed the shoulder of the patient with one hand, and the other hand pressed against the anterior superior iliac spine of the patient. When the patient's waist was rotated to the maximum, both hands were forced at the same time to obliquely pull in the opposite direction (Figure 1). This operation lasted about 5–8 min.

For the first month, treatment was administered twice a week with a 3-day interval and each treatment takes approximately 20 min. After 1 month, the frequency was adjusted to weekly, with an additional weekly session if the pain increased, for 6 months and each treatment takes the same amount of time as before, about 20 min. Treatment was conducted for a total of 7 months.

### Outcome measures and follow-up

The patient received a total of 39 TCMMT treatments within 7 months. There were no significant adverse effects during 7 months of treatment.

Before treatment, after the first TCMMT, 1 month and 7 months of treatment, and 18 months of follow-up, we used ATR and health-related quality of life (HRQOL) for assessment. The HRQOL was assessed using the visual analog scale (VAS) scores (13) and the Scoliosis Research Society-22 (SRS-22) (14) patient questionnaire. The minimum clinically important difference (MCID) (15) was used to assess the effectiveness of clinical measures based on a “responder analysis”. The converted MCID scores (16, 17) in Table 1 were extrapolated from the literature. After 7 months of treatment and 18 months of follow-up, we re-examined the patient's anteroposterior radiographs of the whole spine to assess Cobb angle changes.

**Table 1:** The converted MCID scores in HRQOL.

HRQOL	The converted MCID score
VAS^a^	12
SRS-22^b^	15.62
SRS-22 pain	2
SRS-22 function	3
SRS-22 mental health	2.15
SRS-22 self-image	6.15
SRS-22 satisfaction	2

MCID, minimum clinically important difference; HRQOL, health-related quality of life; VAS, visual analog scale; SRS-22, scoliosis research society-22.

^a^ The VAS score consists of a 100 mm long scale line, the scale corresponds to the pain score, 0 points means “ No pain”, 100 points means “The most severe pain”, the patient marks the corresponding position on the scale line to represent their average pain level.

^b^ The Scoliosis research society-22(SRS-22) score is composed of 5 parts (pain, functional, mental health, self-image and satisfaction) and 22 questions, each of which is scored from 1 to 5. The total scores of SRS-22 range from 20 to 110, with higher scores indicating better quality of life.

**Figure 2:**
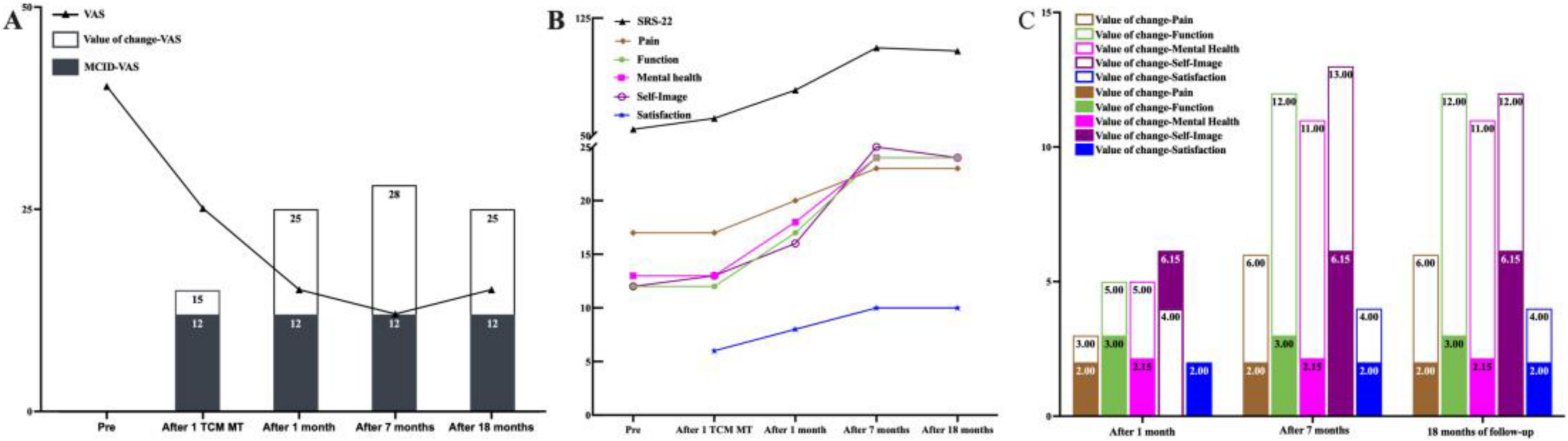
Outcomes of HRQOL before and after treatment and follow-up. **(A)** The patient's VAS score was 40/100 before treatment, 25/100 after the first treatment, 15/100 after 1 month of treatment, 12/100 after 7 months of treatment, and 15/100 at follow-up to 18 months. From the time of receiving the 1st TCMMT treatment until the 18th month after the follow-up, the patients’ VAS change values were higher than the MCID values. **(B)** The patient's SRS-22 score was 54/110 before treatment, 61/110 after the first treatment, 79/110 after 1 month of treatment, 106/110 after 7 months of treatment, and 104/110 at follow-up to 18 months. **(C)** After one month of TCMMT treatment, all SRS-22 results exceeded the preset MCID values, except for SRS-22 Self-Image. At 7 months after treatment and up to 18 months of follow-up, all SRS-22 indices were clinically significant (change values greater than MCID values).

**Figure 3:**
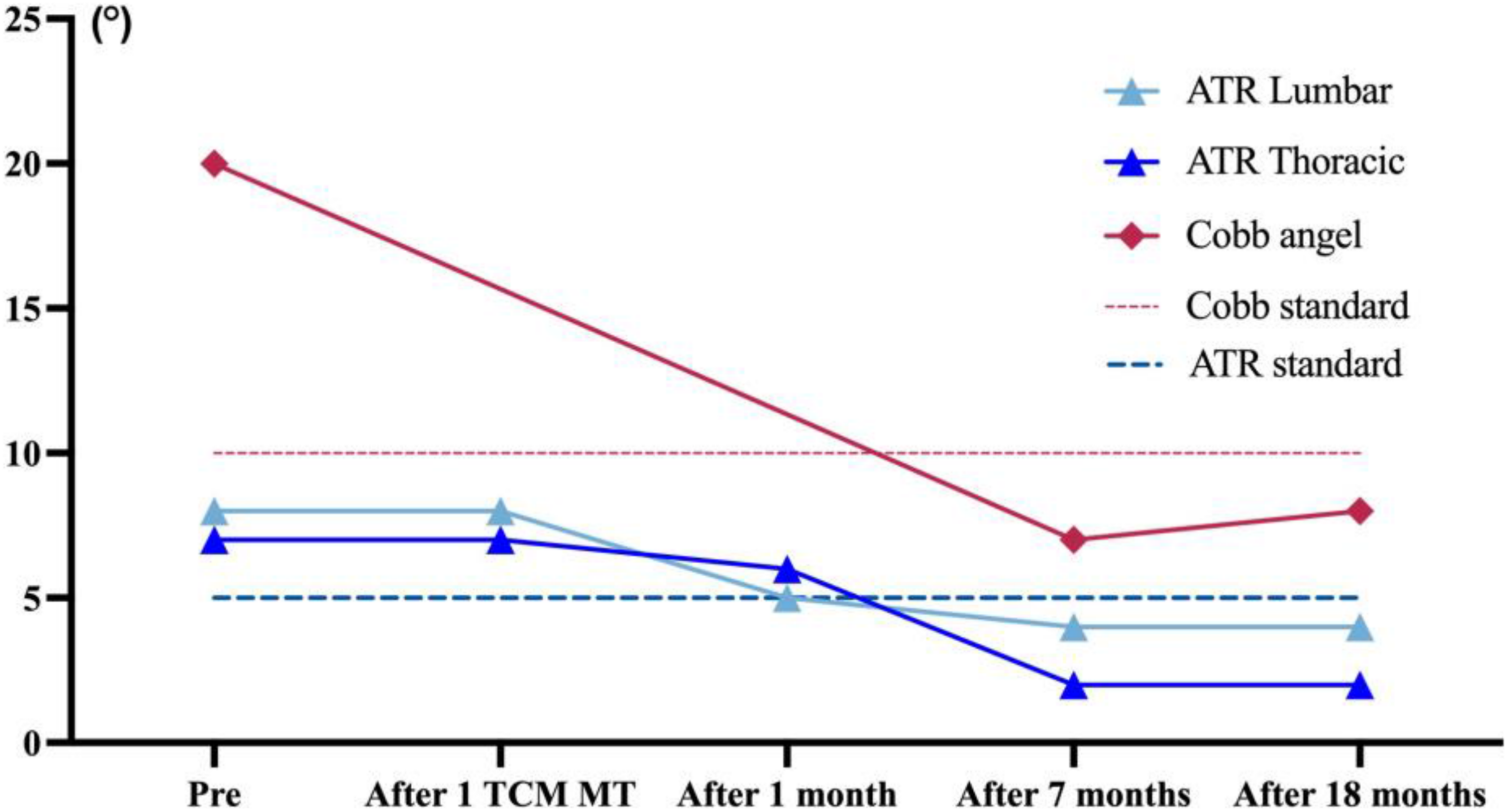
Outcomes of ATR and cobb angle before and after treatment and follow-up. Before treatment, the patient's thoracic ATR angle was 7° and the lumbar ATR angle was 8°, and the Cobb angle was 20°. After 7 months of treatment, the patient's ATR angle and Cobb angle were within the range of normal values until the 18th month of follow-up.

**Figure 4:**
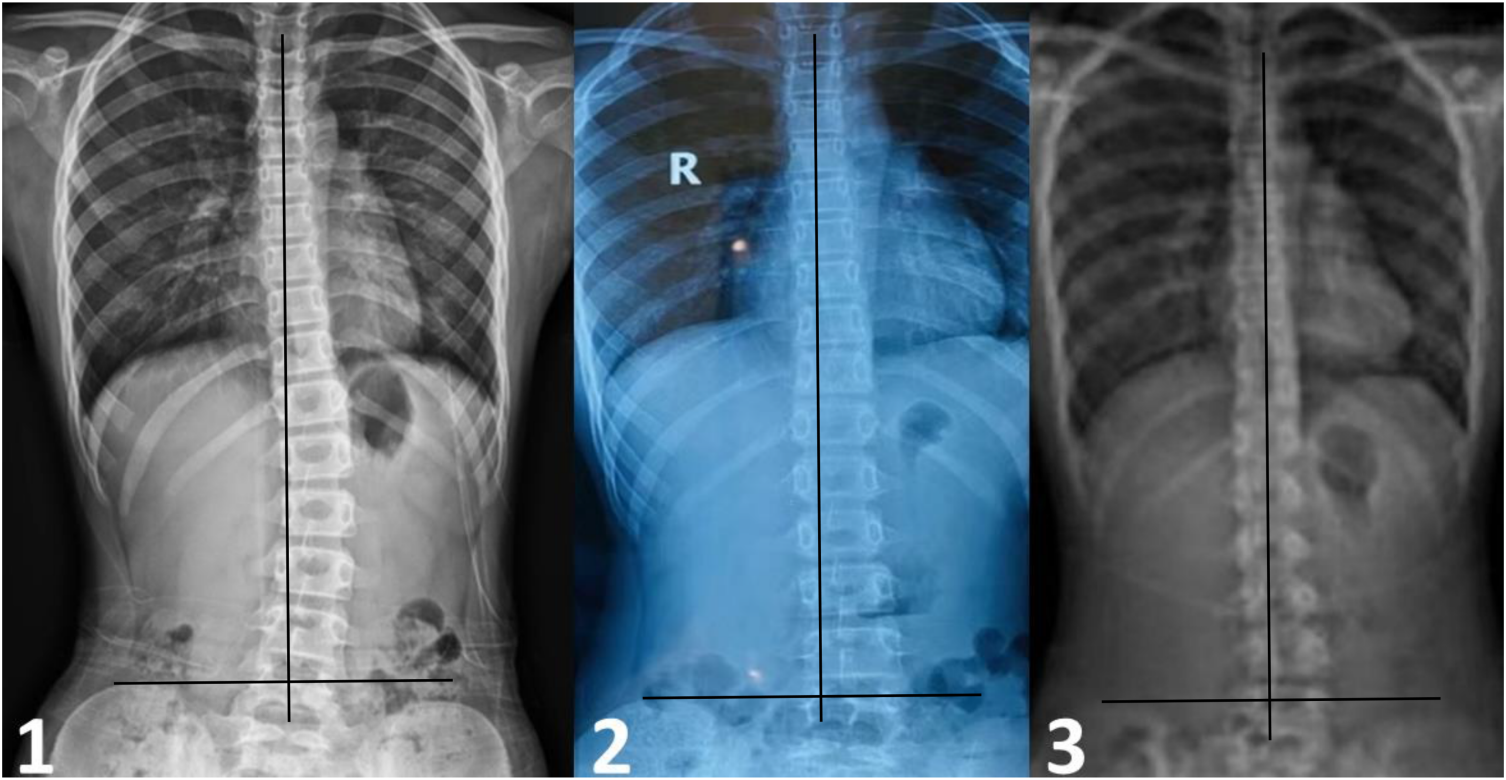
The radiograph of change in the cobb angle during treatment and follow-up. **(1)** Patient was 10 years old. At the first visit, radiographs showed the 20° to the left in lumbar spine scoliosis. **(2)** After 7 months TCMMT treatment, anteroposterior radiographs showing that the Cobb angle was less than 10°, the lumbar spine scoliosis reduced obviously. **(3)** The correction of scoliosis was maintenance stable at 18 months follow-up.

The VAS score decreased from 40 to 25 after the first TCMMT treatment. After 1 month of TCMMT, the VAS score was 15, and the SRS-22 score improved from 54 to 79. The HRQOL results had exceeded MCID values except for the SRS-22 Self-Image scores. The ATR angles for the thoracic and lumbar spine were 6° and 5°, respectively. After 7 months of TCMMT, the patient's VAS score decreased to 12 and the SRS-22 score increased to 106 and the HRQOL results exceeded MCID values. The ATR angle of the thoracic spine decreased to 1.5°, and the ATR of the lumbar spine decreased to 3.5°, and the Cobb angle decreased to 7° from the initial 20°. After 18 months of follow-up, until the patient's first menstrual period was more than 1 year old, and the patient's VAS score was 15 and the SRS-22 score was 104 and the HRQOL results all stayed above the MCID values. The ATR angle at the thoracolumbar segment was maintained at less than 5°, and the Cobb angle was maintained at 10° or less. The results are summarized in Table 2; Figures 2, 3. The Radiograph of changes is in Figure 4.

**Table 2:** Outcomes of HRQOL, ATR and radiographic parameters before and after treatment and follow-up.

Parameters	Pre-treatment	After 1TCMMT treatment	After 1 month of TCMMT	After 7 months of TCMMT	18 months of follow-up
VAS	40	25	15	12	15
SRS-22	54	61	79	106	104
Pain	17	17	20	23	23
Function	12	12	17	24	24
Mental health	13	13	18	24	24
Self-image	12	13	16	25	24
Satisfaction	—	6	8	10	10
ATR					
Thoracic	7°	7°	6°	1.5°	2°
Lumbar	8°	8°	5°	3.5°	4°
Cobb angel	20°	—	—	7°	8°

TCMMT, traditional Chinese medicine manual therapy; HRQOL, health-related quality of life; VAS, visual analog scale; SRS-22, scoliosis research society-22; ATR, axial trunk rotation.

## Discussion

The treatment of MT to solve patients' spine-related pain and negative emotions has its advantages and limitations have been widely described (11). The correction technique for adolescent spinal deformities has achieved considerable methodological support, but the efforts to achieve such correction through the method of TCMMT are relatively silent (18). In our report, TCMMT could reverse the patient's Cobb angle, adjust the patient's back posture, relieve pain, improve quality of life, and achieve more reliable patient satisfaction. Cobb and ATR angle reductions were demonstrated in the results that could be maintained from after 7 months of treatment to 18 months of follow-up. The cost of TCMMT treatment on AIS is far less than the cost of surgery and braces (19, 20). Comparison with MICD through the value of HRQOL changes after treatment can provide patients and policymakers with clearer insights into treatment efficacy, potentially guiding them towards choosing TCMMT. As a safe and effective complementary and alternative therapy, TCMMT may be a new treatment option for patients who choose conservative treatment with benefits for AIS patients. We hope that these findings will offer valuable insights for improving the conservative treatment of AIS in clinical practice.

For AIS patients who choose conservative treatment options, the guidelines (7) may still have room for additional refinement. As patients' demands for aesthetic body shape and spinal health continue to increase, regular observation intervals of several months are likely to make them miss the best treatment window period. Studies have shown that without effective intervention for advanced AIS patients, the curve Angle may increase by an average of 9.6° within 6 months (21, 22). Although bracing and physiotherapeutic scoliosis specific exercise (PSSE) are also the main non-surgical treatments recommended by the guidelines for the treatment of IS, they also limit their use in many cases. For example, wearing braces can affect the patients' appearance, resulting in local skin lesions or pigmentation (23). It is very easy for patients to give up treatment because PSSE requires long periods of daily training for long periods to be effective (24). For those patients who resist PSSE, observation or braces, TCMMT may serve as a viable option to complement the alternative therapies.

There are still areas that need attention in the operation of TCMMT. Firstly, AIS patients whose primary curvature is in the lumbar spine may be more likely to be corrected by TCMMT than those with thoracic curves. For the thoracic spine, the lateral force transmission is more easily absorbed by the costal arch. Since the lumbar spine doesn't have the transverse support of the costal arch, the force will be applied to the lumbar spine directly and produce effectiveness. Secondly, the high frequency and long period of continuous treatment are the keys to the qualitative change in curative effect. Despite reports suggesting, MT can be recommended to improve forward head posture, thoracic kyphosis and pelvic alignment in the short and medium term, but not shoulder posture and scoliosis (25). But our findings suggest otherwise. In our view, by repeatedly adjusting the paraspinal muscle strength and spinal stress on both sides of the spine, TCMMT continuously produces slow remodeling and change of the spine under the effect of reducing the muscle tension on the convex side and increasing it on the concave side. The TCMMT gradually improves the mechanical imbalance of the spine and eventually restores the relative balance of the mechanical properties of the spine and restores the strength of the paravertebral muscles and achieves the treatment goal of delaying the development of scoliosis and correcting the deformity of scoliosis. Finally, it's worth noting that the TCMMT technique may be suitable for correcting mild AIS patients with the Risser sign less than grade 3. This is possibly due to the softer vertebrae of these AIS patients, along with their bones having good growth potential and plasticity.

The evidence of manipulation in the treatment of spinal deformities is still limited (26). Whether TCMMT will show more therapeutic advantages compared with PSSE therapy still needs to be carefully observed. Without enough evidence, clinicians need to be cautious when giving diagnoses and treatment recommendations. We believe that TCMMT remains a recommended alternative treatment option to try for considering conservative treatment strategies. We advocate for future studies that conduct more RCTs to increase the strength of clinical evidence.

### Limitation

The case we reported of scoliosis in which the predominant curvature is in the lumbar segments and is a unilateral curvature of the scoliosis. We are of the opinion that these patients are more amenable to the use of massage therapy. However, in patients with scoliosis in both the thoracic and lumbar spine, the efficacy of manipulative therapy still needs to be carefully observed to ensure robustness of efficacy. In addition, the patient has good treatment compliance and completes all treatments in the course of therapy in this case. The TCMMT requires prolonged and constant stimulation of the paravertebral musculature, and the efficacy of the therapy will probably be inhibited in patients with poor compliance, which is another drawback. Finally, according to the original plan, the patient should be followed up after 6 months, but due to the patient's personal reasons (busy revising for exams), the follow-up is postponed to 7 months after treatment which may lead to potential bias in the results.

## Conclusion

This typical case demonstrates the safe and effective use of TCMMT in the treatment of AIS with LBP and postural abnormalities. After 7 months of TCMMT, the Cobb angle of the main curve was reduced by 13° in AIS patients, which was maintained until the 18-month follow-up. More research is needed to justify its use in the future.

## Data Availability

The original contributions presented in the study are included in the article/Supplementary Material, further inquiries can be directed to the corresponding authors.

## Appendix 1

Health Education Content for adolescent idiopathic scoliosis

1. To educate children and adolescents about the onset, prognosis and progression of scoliosis, to reduce their anxiety and fear of the unknown about scoliosis, and to reduce the psychosocial impact of scoliosis on children and adolescents.
2. Adopt correct body postures, develop good habits of standing, sitting, lying down and writing postures in daily life and study, and avoid prolonged one-shoulder loads and prolonged sitting.
3. Enhance exercise management and exercise the low back muscles. Ensure daily exercise hours and avoid regular exercise programs that are unilaterally dominated or unilaterally powered.
4. Cultivate healthy eating habits among children and adolescents and provide nutritionally balanced meals that are beneficial to bone development.
5. Avoid over-exertion: Patients are advised to avoid standing or sitting for long periods of time, and to get up and move around at regular intervals to relax the body.
